# Association between number of vasopressors and mortality in COVID-19 patients

**DOI:** 10.1016/j.ahjo.2023.100324

**Published:** 2023-09-16

**Authors:** Michael Sunnaa, Mina Kerolos, Max Ruge, Ahmad Gill, Jeanne M. Du-Fay-de-Lavallaz, Perry Rabin, Joanne Michelle Dumlao Gomez, Kim Williams, Anupama Rao, Annabelle Santos Volgman, Karolina Marinescu, Tisha Marie Suboc

**Affiliations:** a Rush University Medical Center, Chicago, IL, United States; b Thomas Jefferson University Hospital, Philadelphia, PA, United States; c University of Nevada Las Vegas, Las Vegas, NV, United States

**Keywords:** COVID-19, Vasopressors, 60-day mortality, Intensive care unit, Shock

## Abstract

**Study objective:**

Study the clinical outcomes associated with the number of concomitant vasopressors used in critically ill COVID-19 patients.

**Design:**

A single-center retrospective cohort study was conducted on patients admitted with COVID-19 to the intensive care unit (ICU) between March and October 2020.

**Setting:**

Rush University Medical Center, United States.

**Participants:**

Adult patients at least 18 years old with COVID-19 with continuous infusion of any vasopressors were included.

**Main outcome measures:**

60-day mortality in COVID-19 patients by the number of concurrent vasopressors received.

**Results:**

A total of 637 patients met our inclusion criteria, of whom 338 (53.1 %) required the support of at least one vasopressor. When compared to patients with no vasopressor requirement, those who required 1 vasopressor (V1) (adjusted odds ratio [aOR] 3.27, 95 % confidence interval (CI) 1.86–5.79, p < 0.01) (n = 137), 2 vasopressors (V2) (aOR 4.71, 95 % CI 2.54–8.77, p < 0.01) (n = 86), 3 vasopressors (V3) (aOR 26.2, 95 % CI 13.35–53.74 p < 0.01) (n = 74), and 4 or 5 vasopressors(V4–5) (aOR 106.38, 95 % CI 39.17–349.93, p < 0.01) (n = 41) were at increased risk of 60-day mortality. In-hospital mortality for patients who received no vasopressors was 6.7 %, 22.6 % for V1, 27.9 % for V2, 62.2 % for V3, and 78 % for V4-V5.

**Conclusion:**

Critically ill patients with COVID-19 requiring vasopressors were associated with significantly higher 60-day mortality.

## 1 Introduction

Coronavirus disease 2019 (COVID-19), the illness caused by severe acute respiratory syndrome coronavirus 2 (SARS-CoV-2), has led to over 6.9 million deaths worldwide as of April 2023 [1]. Shock is characterized by decreased oxygen delivery and/or increased oxygen consumption or inadequate oxygen utilization leading to cellular and tissue hypoxia. It is a life-threatening condition of circulatory failure and most commonly manifested as hypotension [2]. While multiple causes of shock have been identified in COVID-19 patients, distributive shock secondary to sepsis from the virus or bacterial co-infection appears to be the predominant cause of shock [[3], [4], [5]], while cytokine storm has also been associated with COVID-19 [6]. Cardiogenic shock has been reported as well [7]. Other causes of hypotension requiring vasopressors include intubation and sedative medications [8]. A study by Argenziano et al. in 2020 found that 94 % of COVID-19 ICU patients in New York City were receiving vasopressors [9]. Salacup et al. in 2020 showed that 69 % of patients who needed vasopressors suffered inpatient mortality [10]. Between 5 % and 10 % of the patients infected with COVID-19 require intensive care unit admission, with up to 67 % developing shock [11]. The use of vasopressors has also been associated with higher mortality [12]and independently associated with increased odds of intubation [4]. A meta-analysis in 2022 demonstrated that the highest mortality rate in COVID-19 patients receiving vasopressors was with vasopressin or epinephrine, and the lowest with angiotensin II; however, they did not specify the number of vasopressors used nor the mortality with the increasing number of vasopressors used [13]. To our knowledge, there is no data published regarding the specific mortality risk with an increasing number of concurrent vasopressors used in COVID-19.

The overall data on mortality in patients receiving multiple vasopressors is diverse. A 2017 study investigating vasopressor mortality in those with sepsis with the addition of numerous vasopressors showed the number of simultaneous vasopressors and vasopressor dose performed equally well in predicting death or survival. This study concluded that by receiving three or more vasopressors at full dose, 30-day mortality reached 92.3 % (95 % CI: 79.4 %–100.0 %) [14]. However, a 20-year meta-analysis in 2015 found that inotrope and vasopressor therapy is not associated with differences in mortality in the overall population and in most sub settings, defined as 14 different sub settings ranging from sepsis to liver cirrhosis complications [15].

In the light of the above, the purpose of this study is to assess outcomes with vasopressor support in COVID-19 patients.

## 2 Methods

### 2.1 Patients

This was a retrospective cohort study of patients at least 18 years old with COVID-19, confirmed via polymerase chain reaction, March 2020 to October 2020 who were admitted to Rush University System for Health (RUSH), a 664-bed academic medical center. The study time window of March 2020–October 2020 was chosen to capture the first strains of SARS-CoV-2. Patients were included if they were admitted to the intensive care units (ICUs) with continuous vasopressor infusions. Vasopressors included norepinephrine, phenylephrine, vasopressin, dopamine, and epinephrine. Patients receiving vasopressors were categorized into four groups: those receiving 1 vasopressor, 2 concomitant vasopressors, 3 concomitant vasopressors, and those receiving 4 or 5 concomitant vasopressors. These groups were compared to ICU patients who did not receive any vasopressors. Exclusion criteria included patients who received push dose pressors.

### 2.2 Data collection

Data was collected through a combination of automatic and manual extraction methods. The medical record of each patient included in the study was followed and reviewed by physician investigators for a minimum of 60 days from the first day of their COVID-19 admission by chart review of both the RUSH system. In addition, records from local hospitals that use EPIC electronic medical records (EPIC Systems, Verona, WI) were also reviewed.

Comorbidities were defined with automatically extracted data from the International Statistical Classification of Diseases and Related Health Problems, 10th revision (ICD-10) codes.

Patient records were manually reviewed for mortality data. If there was no mortality documented, the patient was counted as alive. Race was self-identified and extracted as such from the medical record. Patients of Hispanic and Latino origin were categorized as “Other” in the medical records and further identified as Hispanic and Latino ethnicity.

Baseline characteristics included age, sex, body mass index (BMI), race, and a variety of comorbidities that were used in multivariable logistic regression (Table 1).

**Table 1:** Baseline characteristics of patients receiving vasopressors.

Max pressors required	0	1	2	3	4 or 5	p-Value
n	299	137	86	74	41	
Age	60.00 [46.00, 73.00]	60.00 [48.00, 70.00]	63.00 [52.00, 69.00]	63.00 [52.00, 70.00]	60.00 [53.00, 66.00]	0.781
Male (%)	175 (58.5)	85 (62.0)	58 (67.4)	45 (60.8)	25 (61.0)	0.676
BMI	30.30 [25.70, 35.50]	32.36 [28.20, 37.80]	30.10 [27.22, 34.48]	34.90 [28.30, 41.72]	32.40 [28.40, 39.20]	<0.001
Race (%)						0.231
White	76 (27.2)	33 (25.4)	20 (24.1)	13 (19.4)	11 (28.9)	
Other	94 (33.7)	56 (43.1)	41 (49.4)	27 (40.3)	15 (39.5)	
Black	109 (39.1)	41 (31.5)	22 (26.5)	27 (40.3)	12 (31.6)	
Comorbidities						
Current smoker (%)	14 (5.3)	7 (6.0)	0 (0.0)	2 (3.3)	0 (0.0)	0.203
Atrial fibrillation (%)	52 (17.4)	30 (21.9)	32 (37.2)	28 (37.8)	10 (24.4)	<0.001
Coronary artery disease (%)	92 (30.8)	55 (40.1)	39 (45.3)	32 (43.2)	19 (46.3)	0.028
Hypertension (%)	192 (64.2)	105 (76.6)	71 (82.6)	51 (68.9)	29 (70.7)	0.007
Chronic kidney disease (%)	88 (29.4)	43 (31.4)	22 (25.6)	38 (51.4)	9 (22.0)	0.002
COPD (%)	29 (9.7)	15 (10.9)	11 (12.8)	9 (12.2)	3 (7.3)	0.849
Diabetes mellitus (%)	149 (49.8)	72 (52.6)	45 (52.3)	47 (63.5)	22 (53.7)	0.346
Asthma (%)	39 (13.0)	24 (17.5)	9 (10.5)	8 (10.8)	3 (7.3)	0.35
Cancer (%)	32 (10.7)	16 (11.7)	14 (16.3)	11 (14.9)	2 (4.9)	0.334
Ventricular arrhythmia (%)	11 (3.7)	12 (8.8)	16 (18.6)	8 (10.8)	4 (9.8)	<0.001
Stroke (%)	62 (20.7)	22 (16.1)	12 (14.0)	12 (16.2)	3 (7.3)	0.187
Acute myocardial infarction (%)	55 (18.4)	43 (31.4)	31 (36.0)	31 (41.9)	15 (36.6)	<0.001
DVT or pulmonary embolism (%)	43 (14.4)	34 (24.8)	25 (29.1)	30 (40.5)	15 (36.6)	<0.001

### 2.3 Outcomes

The outcome of this study was 60-day mortality among patients receiving one or more vasopressors compared to those not receiving vasoactive medications in the ICU.

### 2.4 Statistical analysis

All data analysis, including statistical analyses, was performed using RStudio version 1.3 (Boston, Massachusetts). Continuous variables were compared with *t*-tests or the Wilcoxon rank-sum test, and categorical variables with the Pearson chi-square test. Continuous variables are reported with mean and standard deviation for normally distributed variables and median and interquartile range for variables not normally distributed. Categorical variables are reported as counts and proportions.

Multivariable logistic regression was performed between the comorbidities as predictors for 60-day mortality. Including all the comorbidities into a single model for each outcome allows for assessing each covariate independently by adjusting the other variables in the model. Covariates were chosen after a review of existing literature describing risk factors for adverse outcomes in COVID-19, with preference given to variables with previously significant findings. Adjusted odds ratios (aOR) with 95 % confidence intervals (CI) are reported for logistic regression. The threshold for statistical significance was set to a p-value < 0.05.

## 3 Results

A total of 637 patients met the inclusion criteria. The cohort was predominantly male (60.9 %) with a median age of 60 years old (interquartile range 49–70).

**Fig. 1:**
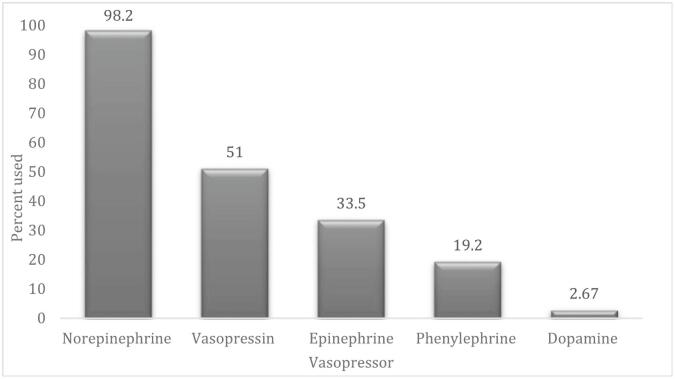
Bar graph showing percentage (y axis) of use of each vasopressor (x axis) of 338 patients who required 1 and/or more vasopressors.

Of the 637 patients included in the study, 338 (53 %) received at least one vasopressor. Norepinephrine (98.2 %) was the most utilized vasopressor followed by vasopressin (51 %), epinephrine (33.5 %), phenylephrine (19.2 %), and dopamine (2.7 %) (Fig. 1). Of those who received vasopressors, the majority (201 patients, 59.5 %) required >1 concurrent vasopressor with 86 patients (25.4 %), 74(21.9 %), and 41(12.1 %) patients requiring 2, 3, and 4–5 concurrent vasopressors respectively.

When stratified by the number of vasopressors required, no significant difference was present in age, gender, or race. In comparing baseline characteristics, those requiring more concurrent vasopressors were generally more likely to have an underlying history of atrial fibrillation (p < 0.001), chronic kidney disease (p < 0.001), history of myocardial infarction (p < 0.001), history of ventricular arrhythmia (p < 0.001), and history of pulmonary embolism or venous thromboembolism (p < 0.001; Table 1).

**Fig. 2:**
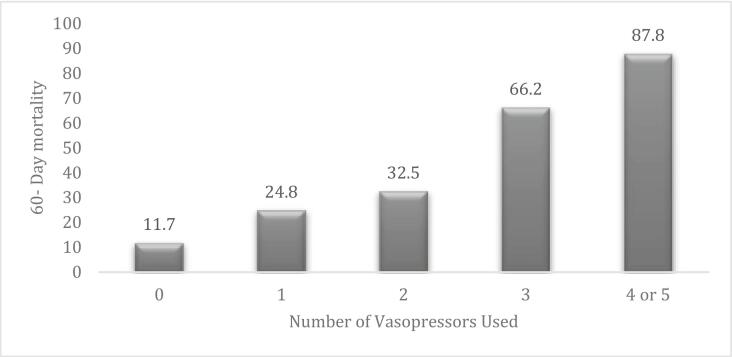
Bar graph showing 60 day mortality (y axis) vs number of vasopressors used (x axis).

**Fig. 3:**
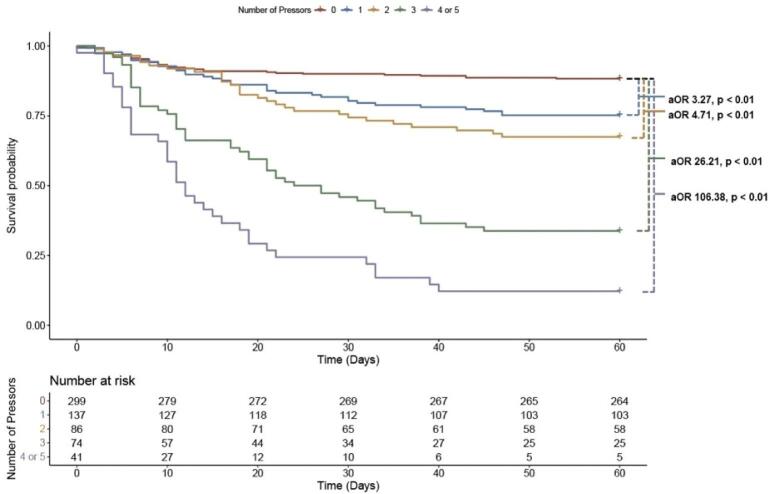
Kaplan-Meier survival curve based on maximal number of concurrent pressors required. Odds ratios are adjusted for age, BMI and history of CAD and diabetes mellitus. Abbreviations: aOR = adjusted odds ratio; BMI = body mass index; CAD = coronary artery disease.

The 60-day mortality rate of the overall cohort was 28.6 %. When stratified by number of pressors, the mortality rates were 11.7 % for 0 pressors (V0), 24.8 % for 1 vasopressor, 32.5 % for 2 vasopressors (V2), 66.2 % for 3 vasopressors (V3), 87.8 % for 4 or 5 vasopressors (V4–5) (Fig. 2). After adjusting for age, body mass index (BMI), coronary artery disease (CAD), and diabetes mellitus, those who required 1 (adjusted OR [aOR] 3.27, 95 % CI 1.86–5.79, p < 0.01), 2 (aOR 4.71, 95 % CI 2.54–8.77, p < 0.01), 3 (aOR 26.21, 95 % CI 13.35–53.74, p < 0.01), and 4 or 5 (aOR 106.38, 95 % CI 39.17–349.93, p < 0.01) vasopressor(s) were at significantly increased risk of 60-day mortality when compared to those with no pressor requirement (Fig. 3).

Those who received vasopressors were significantly more likely to require mechanical ventilation. 34 (11.4 %) patients in the no vasopressor group required mechanical ventilation versus 324 (95.8 %) of those receiving vasopressors (p < 0.001) (Supplemental Table 1).

## 4 Discussion

Among critically ill patients with COVID-19, vasopressor use was associated with significantly higher 60-day mortality compared to the no vasopressor group.

To our knowledge, this is the first study that investigated mortality with addition of vasopressors in COVID-19 patients. Our results showed higher odds of 60-day mortality with all vasopressor groups when compared no vasopressor groups and demonstrates higher odds of mortality with 3,4–5 vasopressors vs 1 or 2 vasopressors. Our study confirms previous observations that vasopressors are associated with increased risk of mortality [10,12]. When compared to a 2017 study regarding vasopressors in septic shock in patients without COVID-19, our study shows that COVID-19 patients also have increased mortality with 3 or more vasopressors [14]. But it is incongruent with a 2015 meta-analysis which showed no increase in mortality with vasopressor administration, however the referenced 2015 study did qualify with “majority of sub settings” [15]. Our results support a 2023 meta-analysis, which showed higher mortality in COVID-19 patients receiving vasopressors [16]. It also supports other studies that show patients on vasopressors have an increased risk of intubation [4]. Compared to the study by Salacup et al., which showed that 69 % of COVID-19 patients who needed vasopressors suffered inpatient mortality [10], our overall 60-day mortality in our cohort was lower at 28.6 % despite being a 60-day period than an in-hospital period.

The baseline characteristics were mostly similar in our patients who required vasopressors and those who did not. However, patients who required vasopressors were more likely to be obese, have a prior history of atrial fibrillation, ventricular arrhythmia, hypertension, coronary artery disease, history of acute myocardial infarction, chronic kidney disease, deep venous thromboembolism, or pulmonary embolism. However, age, race, and comorbidities such as smoking, chronic obstructive pulmonary disease (COPD), asthma, diabetes mellitus, cancer or stroke were not associated with an increased risk of requiring vasopressors (Table 1).

Obese COVID-19 patients in the ICU requiring vasopressor support significantly more often than non-obese COVID-19 ICU patients could be explained by several mechanisms; impairment of immune system, pro-inflammatory state, and adipose tissue could be a reservoir for the SARS-Cov2 Virus. In obesity, anti-inflammatory M2 type macrophages are switched to M1 pro inflammatory macrophages [17,18]. Adiponectin can also stimulate anti-inflammatory cytokines [19]. Furthermore, obese patients often have lower systemic vascular resistance [20]. The increased risk of arrhythmias could partly be explained by the fact that patients in the vasopressor subgroups already had higher prevalence of atrial fibrillation and ventricular arrhythmia. These patients are predisposed to the arrhythmogenic nature of vasopressors [21]. Patients with coronary artery disease are at increased risk of vasopressor administration possibly due to a secondary component of decreased systolic function, and further supported by patients with a history of acute myocardial infarction could be developing increased cardiogenic shock, due to COVID-19 effects of the virus on endothelial cells and increased propensity for vascular thrombosis [22]. Lastly, chronic kidney disease patients more likely requiring pressors is congruent with a 2022 review highlighting poorer outcomes in chronic kidney disease patients in the ICU [23]. Furthermore, the increased mechanical ventilation in the vasopressor group could be explained by more severe disease requiring both interventions of vasopressors and mechanical ventilation.

The strength of this study is homogenous population, sample size, 60-day outcomes for the study to provide ample time to assess mortality from COVID-19 infection, and multivariable logistic regression to help correlation between independent and dependent variables.

Our study has limitations by the nature of the pandemic crisis. The data was obtained from March to October 2020, which limits the scope of the paper to the earliest COVID-19 strains, but this was a time period which allowed us to focus on the first strains of COVID-19 infections. Next, the patients were not stratified by classifications of shock, rather simply grouped by ICU patients with COVID-19 who received vasopressors versus those who did not. This was due to the limited diagnostic tools during the COVID-19 pandemic, and we did not have the Swann Ganz Catheter in all patients to delineate the types of shock. Also, the classification of exposure by the number of vasopressors has the inherent ability to over/underestimate accurate vasopressor exposure. In our study, the dosing of the vasopressors was not studied due to the titration nature of vasopressors and the limited access to maximum doses in the medical chart.

Other limitations of this study are the retrospective design of the study, the lack of clarity regarding initiation of vasopressors for shock versus isolated hypotension, single-center study, and the use of electronic health records have inherent sources for error; in addition, there are potential confounders for the study, timing of vasopressor initiation and dosing.

Further studies investigating more precise quantification of vasopressor exposure and criteria in starting of additional vasopressors, consideration of the different outcomes in different types of shock (distributive, cardiogenic, etc.) secondary to COVID-19, addition of inotropes, and data from other COVID-19 strain surges, are needed for a further understanding of vasopressors in COVID-19 patients.

## 5 Conclusion

Critically ill patients with COVID-19 requiring vasopressors were associated with significantly higher 60-day mortality.

The following is the supplementary data related to this article.

Supplemental Table 1 Number of patients requiring mechanical ventilation by respective vasopressor group. Supplemental Table 1

## Ethics statement

Institutional Review Board Protocol was approved according to institution guidelines. No informed consent was obtained as this was a retrospective study.

## Declaration of competing interest

The authors declare that they have no known competing financial interests or personal relationships that could have appeared to influence the work reported in this paper.

## References

[1] World Health Organization Who coronavirus (COVID-19) dashboard. https://covid19.who.int/.

[2] Vincent J.L., De Backer D. (2014). Circulatory shock. N. Engl. J. Med..

[3] Zhang C., Wu Z., Li J.W., Zhao H., Wang G.Q. (2020). Cytokine release syndrome in severe COVID-19: interleukin-6 receptor antagonist tocilizumab may be the key to reduce mortality. Int. J. Antimicrob. Agents.

[4] Rojas-Marte G., Hashmi A.T., Khalid M., Chukwuka N., Fogel J., Munoz-Martinez A., Shani J. (2021). Outcomes in patients with COVID-19 disease and high oxygen requirements. J. Clin. Med. Res..

[5] Varshney A.S., Omar W.A., Goodrich E.L., Bhatt A.S., Wolley A.E., Gong J., Bohula E.A. (2021). Epidemiology of cardiogenic shock in hospitalized adults with COVID-19: a report from the American Heart Association COVID-19 Cardiovascular Disease Registry. Circ. Heart Fail..

[6] Ye Q., Wang B., Mao J. (2020). The pathogenesis and treatment of the ‘Cytokine Storm’ in COVID-19. J. Infect..

[7] Chau V.Q., Giustino G., Mahmood K., Oliveros E., Neibart E., Oloomi M., Mancini D.M. (2020). Cardiogenic shock and hyperinflammatory syndrome in young males with COVID-19. Circ. Heart Fail..

[8] Yao W., Wang T., Jiang B. (2020). Emergency tracheal intubation in 202 patients with COVID-19 in Wuhan, China: lessons learnt and international expert recommendations. Br. J. Anaesth..

[9] Argenziano M.G., Bruce S.L., Slater C.L., Upadhyay S. (2020). Characterization and clinical course of 1000 patients with coronavirus disease 2019 in New York: retrospective case series. BMJ.

[10] Salacup G. (2020). Characteristics and clinical outcomes of COVID-19 patients in an underserved-inner city population: a single tertiary center cohort. J. Med. Virol..

[11] Alhazzani W., Møller M.H., Arabi Y.M., Surviving Sepsis Campaign Guidelines Panel (2020). Surviving sepsis campaign: guidelines on the management of critically ill adults with coronavirus disease 2019 (COVID-19). Crit. Care Med..

[12] COVID-ICU Group on behalf of the REVA Network and the COVID-ICU Investigators (2021). Clinical characteristics and day-90 outcomes of 4244 critically ill adults with COVID-19: a prospective cohort study. Intensive Care Med..

[13] Mermiri M., Mavrovounis G., Laou E., Chalkias A. (2022). Association of vasopressors with mortality in critically ill patients with COVID-19: a systematic review and meta-analysis. medRxiv.

[14] Brand D.A., Patrick P.A., Berger J.T., Spiegler P. (2017). Intensity of vasopressor therapy for septic shock and the risk of in-hospital death. J. Pain Symptom Manag..

[15] Belletti A., Castro M.L., Silvetti S., Landoni G. (2015). The effect of inotropes and vasopressors on mortality: a meta-analysis of randomized clinical trials. Br. J. Anaesth..

[16] Mermiri M., Mavrovounis G., Laou E., Papagiannakis N. (2023). Association of vasopressors with mortality in critically ill patients with COVID-19: a systematic review and meta-analysis. Anesthesiol. Perioper. Sci..

[17] Russo L., Lumeng C.N. (2018). Properties and functions of adipose tissue macrophages in obesity. Immunology.

[18] Hornung F., Rogal J., Loskill P., Löffler B., Deinhardt-Emmer S. (2021). The inflammatory profile of obesity and the role on pulmonary bacterial and viral infections. Int. J. Mol. Sci..

[19] Kumada M., Kihara S., Ouchi N., Matsuzawa Y. (2004). Adiponectin specifically increased tissue inhibitor of metalloproteinase-1 through interleukin-10 expression in human macrophages. Circulation.

[20] Lavie C.J., Milani R.V., Ventura H.O. (2009). Obesity and cardiovascular disease: risk factor, paradox, and impact of weight loss. J. Am. Coll. Cardiol..

[21] Tisdale J.E., Patel R.V., Webb C.R., Borzak S., Zarowitz B.J. (1995). Proarrhythmic effects of intravenous vasopressors. Ann. Pharmacother..

[22] De Luca G., Verdoia M., Cercek M., Dudek D. (2020). Impact of COVID-19 pandemic on mechanical reperfusion for patients with STEMI. J. Am. Coll. Cardiol..

[23] Jdiaa S.S., Mansour R., El Alayli A., Gautam A., Thomas P., Mustafa R.A. (2022). COVID-19 and chronic kidney disease: an updated overview of reviews. J. Nephrol..

