# Supplementary material for: Association between number of vasopressors and mortality in COVID-19 patients
Source: Am Heart J Plus. 2023 Sep 16;34:100324. doi: 10.1016/j.ahjo.2023.100324 (PMC10946008; doi:10.1016/j.ahjo.2023.100324)
Supplement: Supplemental Table 1 — Number of patients requiring mechanical ventilation by respective vasopressor group. [file mmc1.docx]

Supplemental Table 1

| **Max Number of Pressors** | **0** | **1** | **2** | **3** | **4 or 5** | **p -value** |
| --- | --- | --- | --- | --- | --- | --- |
| n | 299 | 137 | 86 | 74 | 41 |  |
| Mechanical Ventilation (%) | 34 ( 11.4) | 131 ( 95.6) | 83 ( 96.5) | 70 ( 94.6) | 40 ( 97.6) | <0.001 |
